# Exploring the Causal Relationship Between Sex Hormones and Depression by Means of Two-Sample Mendelian Randomization Analysis

**DOI:** 10.62641/aep.v54i4.2093

**Published:** 2026-08-15

**Authors:** He Gao, Xiangju Du, Yuncui Huo, Hong Pan, Yongming Xu, Yanbin Hou

**Affiliations:** ^1^Department of Psychiatry, The Affiliated Kangning Hospital of Ningbo University, 305010 Ningbo, Zhejiang, China; ^2^Department of Psychosomatics, The First Affiliated Hospital of Ningbo University, 305010 Ningbo, Zhejiang, China

**Keywords:** sex hormones, depression, mendelian randomization, GWAS

## Abstract

**Background:**

To investigate the potential causal association between sex hormones and depression, a two-sample mendelian randomization (MR) analysis was conducted.

**Methods:**

Summary statistics from Genome-wide Association Study (GWAS) on sex hormones and depression were collected. Sex-specific instruments were used to analyze seven sex hormones, including progesterone (PROG) and bioavailable testosterone (BAT). The inverse variance weighted (IVW) method was employed as the primary analysis, and sensitivity analyses were conducted to assess the robustness of the findings.

**Results:**

The IVW analysis revealed genetically significant association between PROG and depression (odds ratio (OR): 0.95, 95% confidence interval (CI): 0.92–0.98, *p* = 0.002), which remained significant after Bonferroni correction (*p* < 0.0036). A nominally significant association was observed for BAT (OR: 0.90, 95% CI: 0.82–1.00, *p* = 0.049) and depression; however, this association did not survive Bonferroni correction. Upon stratification by gender, these associations were no longer significant (*p* > 0.05). Furthermore, no substantial associations were observed between depression and other sex hormones, including total testosterone (TT), estradiol (E2), follicle-stimulating hormone, prolactin, and luteinizing hormone (*p* > 0.05). Leave-one-out analyses and funnel plots (indicating balanced pleiotropy), confirmed the reliability of these findings, supporting the robustness of the results. Significant heterogeneity was observed in seven exposures: E2_Female, TT_Male, TT_Both, BAT_Both, BAT_Male, BAT_Female, and TT_Female. Additionally, Mendelian Randomization Pleiotropy Residual Sum and Outlier (MR-PRESSO) analysis identified outliers for BAT_Both, BAT_Female, E2_Female, TT_Both, and TT_Female; however, excluding these outliers did not alter the results.

**Conclusions:**

This study indicates a potential causal association between genetically predicted PROG levels and a decreased risk of depression. While BAT also suggested a potential protective effect, this association was considered suggestive after multiple testing correction and requires further validation.

## Introduction

Depression is a common mental health disorder that greatly impairs a person’s quality of life. Its main characteristics include persistent negative mood, anhedonia, and a range of cognitive and physical symptoms [1]. The prevalence of depression was found to be 3.2% among the 245,404 participants from 60 countries [2]. A previous meta-analysis reported that women are approximately twice as likely as men to experience depression (roughly 6% vs. 4%); this gender gap typically emerges during puberty and remains prominent throughout the reproductive years, suggesting that biological factors, especially sex hormones, may play a crucial role in this increased susceptibility [3]. The etiology of depression was multifaceted, involving neurobiological, genetic, epigenetic, psychosocial, and environmental factors [4, 5, 6]. Understanding the etiology of depression is essential for developing effective treatment and prevention strategies.

Sex hormones, including bioavailable testosterone (BAT), and total testosterone (TT), play a key role in regulating reproduction and all sorts of various bodily processes [7]. These hormones are known to influence mood and behavior, and their dysregulation has been found to be associated with the onset and progression of depression. Recent observational studies have highlighted a strong link between sex hormones and depression [8]. Symptoms of depression increased significantly during the menopausal transition and decreased after menopause, with hormone fluctuations contributing to mood changes during this period [9]. A rapid increase in FSH levels was linked to a decreased likelihood of depressive symptoms post-menopause [9]. Lower estradiol concentrations are often associated with an elevated risk of depression, particularly in peri-menopausal and post-menopausal women [9]. Researchers such as Joffe reported that postmenopausal women with large fluctuations in estradiol levels and a lack of progesterone (PROG) during ovulation were associated with higher levels of depressive symptoms, suggesting that hormonal dysregulation during the menopause transition contributes to mood instability [10]. McIntyre *et al*. [11] suggested that lower levels of BAT and TT in middle-aged men are significantly associated with depressive symptoms. This association was more pronounced in men with major depressive disorder. Low TT levels were weakly linked to appetite problems in male subjects, while extremely higher levels are correlated with sleep problems and mild fatigue; however, no consistent symptom-specific associations were observed in women [12]. Although there have been numerous prospective investigations exploring the relationship between sex hormones and depression, their findings may be confounded by various variables, complicating the determination of definitive causal associations.

In this study, we have adopted a sophisticated statistical method, mendelian randomization (MR) techniques, to examine the potential causality between various exposures and health outcomes [13]. The basic theory of MR relies on the random combination of genetic variants occurring naturally at conception [14]. By helping investigators surmount the inherent limitations of standard observational studies, MR analysis enhances the efficiency and credibility of potential causal associations in these studies [15].

This research delves deeper into the role sex hormones play in the onset and development of depression through a two-sample MR analysis. This study assumes there’s a causal link between sex hormones and the risk of depression.

## Methods

### Study Design

**Fig. 1.**
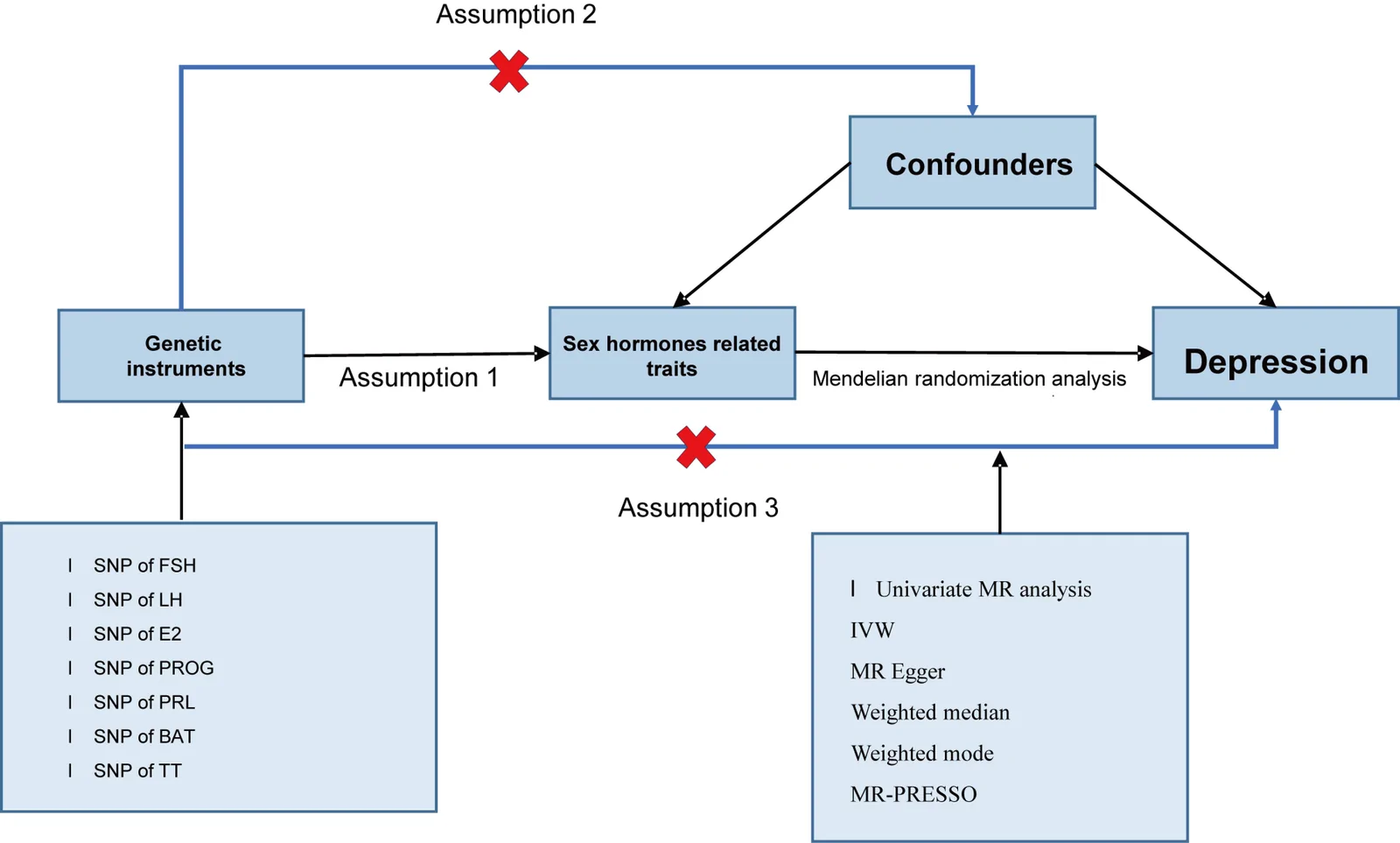
**Flowchart of the mendelian randomization (MR) study**. Assumption 1 indicates that sex hormones significantly correlate with genetic instrumental variables (IVs). Assumption 2 asserts that these IVs do not influence outcomes via confounding variables. Assumption 3 suggests that genetic IVs do not directly affect depression outcomes but do so only through indirect exposure to sex hormones. Abbreviations: IVW, Inverse variance weighted; MR, mendelian randomization; PROG, Progesterone; SNP, Single nucleotide polymorphism.

In this study, the two-sample MR analysis was employed to explore the potential causal association between sex hormones and depression. This investigation was based on aggregated genetic data from relevant genome-wide association studies (GWAS). It is worth noting that MR analysis relies on three basic assumptions: there is a strong association between genetically determined variation and sex hormones; the genetic variants should not be associated with any confounders of the exposure-outcome relationship; and the selected genetically determined variation has a unique effect on the risk of depression through sex hormones (Fig. 1). This MR study utilized openly accessible databases, including FinnGen R10 for depression, the European Bioinformatics Institute (EBI) GWAS Catalog for most sex hormone exposures, and the Leipzig Health Atlas for PROG. Therefore, it did not require ethical committee clearance or informed consent from participants.

### Data Source

The datasets for sex hormones and depression utilized in this MR analysis were sourced from publicly available summary data of the largest GWAS. The selection of these specific sex hormones—including BAT, follicle-stimulating hormone (FSH), progesterone (PROG), estradiol (E2), prolactin (PRL), luteinizing hormone (LH), and total testosterone (TT)—was primarily determined by the availability of high-quality, large-scale GWAS summary statistics in public repositories. These hormones represent the most commonly studied sex-related steroids and proteins, with sufficient genetic data to ensure the reliability and statistical power of the MR analysis. The data of depression was sourced from the FinnGen collaboration, with diagnoses defined according to the International Classification of Diseases, 10th Revision (ICD-10) codes F32 (depressive episode) and F33 (recurrent depressive disorder) [16].

To ensure the specificity of the case-control comparison, individuals with any mood (affective) disorders—including manic episodes (F30), bipolar affective disorder (F31), persistent mood disorders (F34), and other unspecified mood disorders (F38, F39)—were excluded from the control group. The GWAS data for sex hormones, such as BAT, FSH, PROG, E2, PRL, LH, and TT, were utilized, with detailed information provided in **Supplementary Table 1**. To ensure the validity of the MR assumptions and minimize bias from sample overlap, we meticulously verified the independence of the exposure and outcome cohorts. The depression data was obtained from the FinnGen R10 release, composed of an isolated Finnish population. In contrast, the genetic associations for sex hormone exposures were primarily derived from the UK Biobank (for BAT, TT, and E2) and other specific European studies (such as the Leipzig Health Atlas for PROG, which utilized cohorts from Leipzig, Germany, and the INTERVAL study for FSH and LH). Given the distinct geographic and genetic backgrounds of these cohorts, the samples used to estimate the associations between exposure and outcome are independent, with no significant sample overlap. Detailed information on GWAS studies used in this MR analysis is presented in **Supplementary Table 1**. The data for FSH and LH were sourced from the study by Sun* et al*. [17], which reported the genomic landscape of the plasma proteome in humans and included 3301 participants from Europe. The data source for E2 was obtained from the study by Schmitz *et al*. [18], including 163,985 females and 147,690 males. The PROG GWAS summary statistics were obtained from the Leipzig Health Atlas, including sex-specific datasets comprising 1358 males and 1261 females, as well as a combined dataset including 2070 European participants [19]. The data for PRL come from research conducted by Folkersen *et al*. [20], which included 21,758 European cases. The Ruth *et al*. [21] study, which revealed sex-specific impacts of testosterone levels, provided the data for BAT and TT (**Supplementary Table 1**).

### Instrumental Variable (IV) Selection

To investigate the potential causal association between sex hormones and depression, IVs were selected based on multiple criteria. Specifically, SNPs significantly associated with exposures, including FSH_Both, LH_Both, PROG_Both, PROG_Male, PROG_Female, E2_Female, and PRL_Both were selected with a *p*-value < 5 × 10^-6^ due to the limited number of SNPs. For other exposures, SNPs were selected with a stricter *p*-value < 5 × 10^-8^ [22]. In cases where fewer than three SNPs were identified at the conventional threshold (*p* < 5 × 10^-8^), such as for PROG and FSH, the selection criteria were relaxed to *p* < 5 × 10^-6^ to ensure a sufficient number of instrumental variables (IVs) for analysis. This approach is methodologically acceptable, provided that the instruments are sufficiently strong. Burgess *et al*. [23] (2013) noted that a threshold of *p* < 1 × 10^-5^ can be utilized to maintain statistical power while minimizing weak instrument bias. To further validate this, we calculated the F-statistic for all IVs; all selected SNPs exhibited an F-statistic > 10 (ranging from 20.87 to 1409.01), confirming the robustness of these variants in predicting the exposures. Secondly, to ensure sufficient genetic diversity among IVs, we included SNPs with a minor allele frequency (MAF) higher than 0.01 [24]. Thirdly, to minimize the effects of linkage disequilibrium (LD), SNPs were clumped using an R^2^ threshold of < 0.001 and a clumping window of 10,000 kb [25]. Fourthly, when the selected IVs did not appear in the depression summary data, we identified alternative SNPs with high linkage disequilibrium (R^2^> 0.8) and used them as surrogate variables to ensure the validity of the analysis [22]. Fifth, for consistency, the datasets related to sex hormones and depression were standardized by eliminating ambiguities in SNPs with inconsistent allele and minor allele frequencies. These SNPs were selected as the instrumental variables (IVs) for the MR analysis [26].

### Statistical Analysis

All statistical analyses were performed using R software (version 4.0.5, R Foundation for Statistical Computing, Vienna, Austria) with the “TwoSampleMR” and “MR-PRESSO” packages. To ensure the robustness of the genetic instruments, the power of each SNP as an instrumental variable was evaluated using the F-statistic. This value is derived through the following formula: F = R^2^*(N-2)/(1-R^2^), in which R^2^ indicates the percentage of variation in exposure attributable to SNPs, and N denotes the sample size [24]. The F-statistics for SNPs exceeding 10 were considered sufficiently strong and were incorporated into our analysis.

The Inverse variance weighted (IVW) method was utilized as the primary analysis to estimate causal effects, supplemented by MR-Egger, weighted median, and weighted mode analyses for sensitivity testing [25, 27, 28]. For each exposure-outcome relationship, odds ratios (OR) and 95% confidence intervals (CI) were calculated. The IVW method relies on the principle that IVs with lower variance exert a greater influence on the overall effect estimate compared to those with higher variance [29]. To clearly interpret the findings, the data were analyzed using visualization techniques, including a scatter plot and sensitivity analysis chart [30]. A corrected *p*-value threshold of < 0.0036 (0.05/14) was considered statistically significant. Results with a raw *p* < 0.05 but ≥ 0.0036 were considered nominally significant and should be interpreted with caution.

To ensure the robustness of the conclusions when results from different methods were inconsistent, the following criteria were adopted: the IVW result was primarily referenced as the main effect estimate. Heterogeneity among the instrumental variables (IVs) was assessed via Cochran’s Q test; if significant heterogeneity was detected (*p* < 0.05), the random-effects IVW model was employed to provide a more conservative estimate. Horizontal pleiotropy was evaluated using the MR-Egger intercept test and the MR-PRESSO global test. If significant horizontal pleiotropy was observed (MR-Egger intercept *p* < 0.05), the results from MR-Egger or the weighted median method were prioritized, as these methods provide more reliable estimates in the presence of pleiotropic variants. Outliers identified by MR-PRESSO (*p* < 0.05) were excluded, and causal estimates were re-evaluated. A leave-one-out analysis was further conducted to ensure that no single SNP disproportionately influenced the results. To control for multiple testing, a Bonferroni correction was applied for the 14 independent exposure-outcome tests.

## Results

### SNPs Selection

For sex hormones including BAT, LH, FSH, PROG, PRL, E2, and TT, a total of 1090 relevant IVs were selected. It is worth noting that the average F-statistic of all these instrumental variables is 77.38, ranging from 20.87 to 1409.01 (see **Supplementary Table 2**). SNPs that were not matched in the depression summary data were replaced with proxy SNPs, as shown in **Supplementary Table 3**.

### MR Analysis Results

**Fig. 2.**
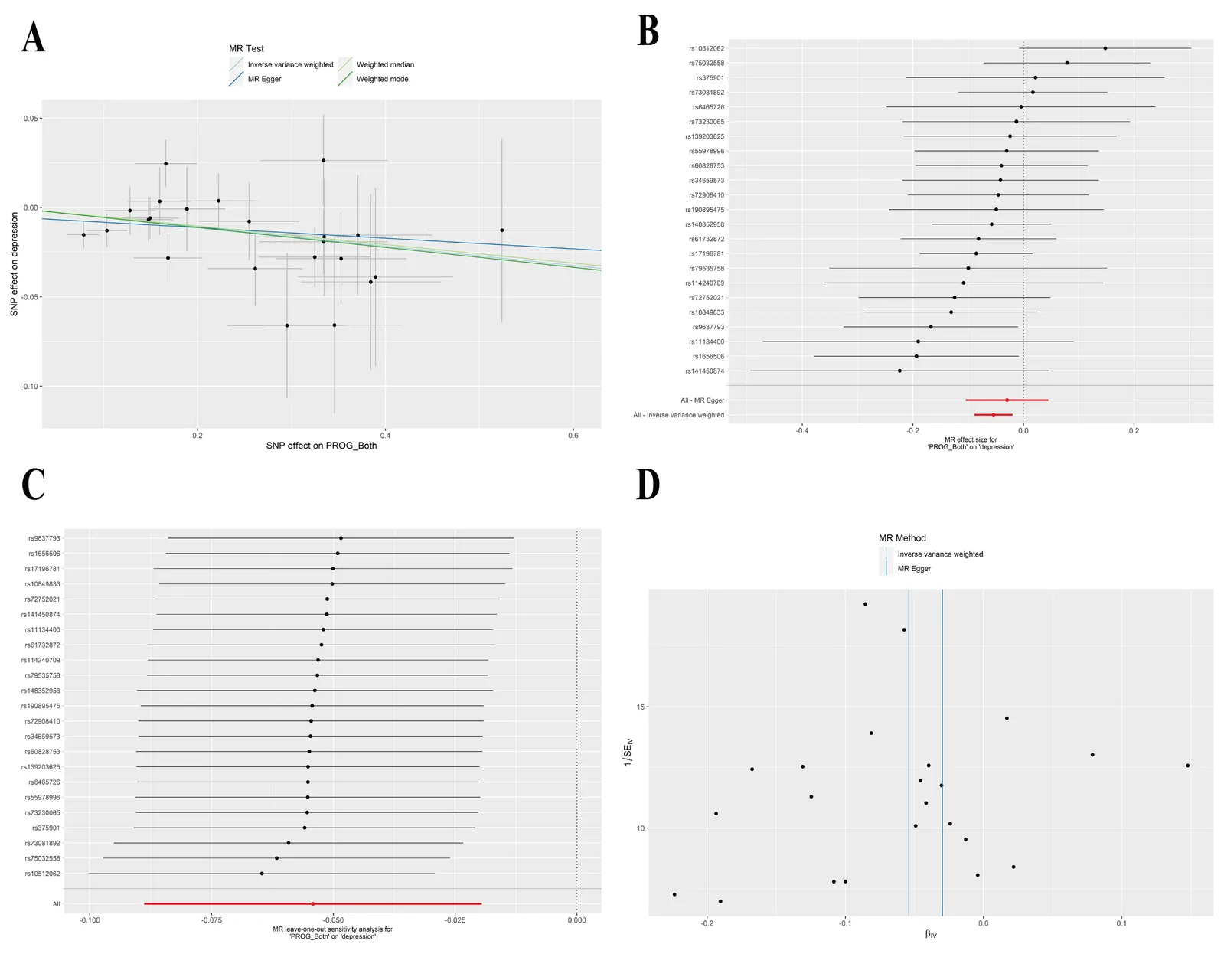
**The MR analysis for the potential causal association between PROG and depression**. (A) A scatter plot depicting the association between PROG and depression. (B) A forest plot presenting the MR estimate. (C) Leave-one-out analysis validating the robustness of the findings. (D) A funnel plot evaluating consistency and potential heterogeneity. PROG, Progesterone; MR, mendelian Randomization.

**Fig. 3.**
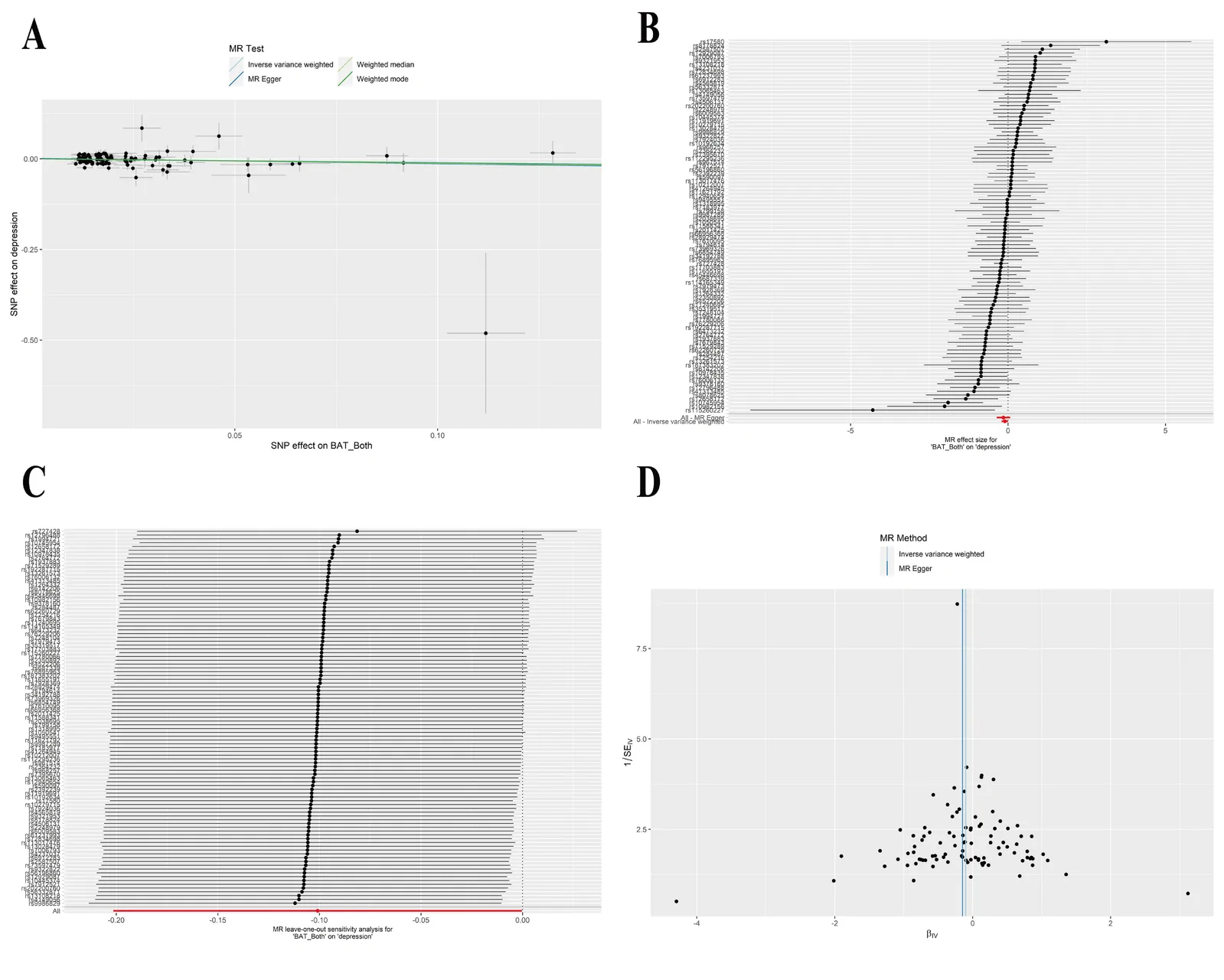
**The MR analysis for the potential causal association between BAT and depression**. (A) A scatter plot depicting the association between BAT and depression. (B) A forest plot presenting the MR estimate. (C) Leave-one-out analysis validating the robustness of the findings. (D) A funnel plot evaluating consistency and potential heterogeneity. BAT, Bioavailable Testosterone; MR, mendelian randomization.

The IVW analyses indicated a strong association between PROG_Both and depression (OR: 0.95, 95% CI: 0.92–0.98, *p* = 0.002, Table 1). The scatter and forest plots further illustrated this strong association between PROG_Both and depression (Fig. 2A and 2B). Additionally, weighted median analysis also indicated a significant association (OR: 0.95, 95% CI: 0.90–1.00, *p* = 0.036, Table 1). After applying the strict Bonferroni correction for the 14 exposure-outcome tests (threshold *p* < 0.0036), the causal association between PROG and depression remained statistically significant (*p* = 0.002). However, the association between BAT and depression, while nominally significant in the IVW analysis (OR: 0.90, 95% CI: 0.82–1.00, *p* = 0.049), was not statistically significant after applying the Bonferroni correction (*p* > 0.0036). Therefore, the relationship between BAT and depression is considered to be suggestive and should be interpreted with caution. Notably, these associations did not maintain statistical significance upon sex-stratified analysis (*p* > 0.05). This divergence from the overall results likely reflects the diminished statistical power within the smaller, sex-specific subgroups rather than a true absence of effect. Specific differences in results between genders were observed: for example, the protective trend of BAT appeared to be more pronounced in females (OR: 0.94, *p* = 0.056) than in males (OR: 1.00, *p* = 0.890), although neither reached statistical significance. This suggests that the overall significant findings may be driven by specific subgroups or that the combined sample provided the necessary power to detect an effect that sex-stratified samples could not. Similarly, the IVW analysis revealed no link between PROG and depression in females (OR: 1.01, 95% CI: 0.99–1.02, *p* = 0.345, Table 1), suggesting that PROG levels do not significantly impact the risk of depression in women. The IVW analysis indicated a significant association between BAT_Both and depression (OR: 0.90, 95% CI: 0.82–1.00, *p* = 0.049) (Table 1, Fig. 3A and 3B). However, supplementary analyses suggested no significant association (Table 1). The IVW analysis showed no significant association between BAT and depression in males (OR: 1.00, 95% CI: 0.94–1.08, *p* = 0.890, Table 1). The IVW analysis results indicate that there is no statistically significant association between BAT and depression in females (OR: 0.94, 95% CI: 0.88–1.00, *p* = 0.056, Table 1). These results suggest that BAT levels do not significantly impact the risk of depression when stratifying by gender.

**Table 1.** **Association between genetically predicted sex hormones and risk of depression**.

Exposure	N.SNP	Methods	OR (95% CI)	*p*-value
PROG_Both	23	IVW	0.95 ( 0.92–0.98 )	0.002
PROG_Both	23	MR Egger	0.97 ( 0.90–1.05 )	0.443
PROG_Both	23	Weighted median	0.95 ( 0.90–1.00 )	0.036
PROG_Both	23	Weighted mode	0.95 ( 0.88–1.02 )	0.155
PROG_Male	7	IVW	0.96 ( 0.88–1.06 )	0.446
PROG_Male	7	MR Egger	0.91 ( 0.68–1.22 )	0.553
PROG_Male	7	Weighted median	1.00 ( 0.90–1.10 )	0.928
PROG_Male	7	Weighted mode	0.99 ( 0.85–1.16 )	0.950
PROG_Female	31	IVW	1.01 ( 0.99–1.02 )	0.345
PROG_Female	31	MR Egger	0.99 ( 0.95–1.03 )	0.567
PROG_Female	31	Weighted median	1.00 ( 0.98–1.02 )	0.868
PROG_Female	31	Weighted mode	0.99 ( 0.95–1.03 )	0.654
BAT_Both	99	IVW	0.90 ( 0.82–1.00 )	0.049
BAT_Both	99	MR Egger	0.86 ( 0.70–1.07 )	0.182
BAT_Both	99	Weighted median	0.88 ( 0.77–1.02 )	0.084
BAT_Both	99	Weighted mode	0.89 ( 0.75–1.07 )	0.220
BAT_Male	83	IVW	1.00 ( 0.94–1.08 )	0.890
BAT_Male	83	MR Egger	0.92 ( 0.80–1.07 )	0.275
BAT_Male	83	Weighted median	1.05 ( 0.95–1.15 )	0.329
BAT_Male	83	Weighted mode	1.07 ( 0.94–1.21 )	0.324
BAT_Female	132	IVW	0.94 ( 0.88–1.00 )	0.056
BAT_Female	132	MR Egger	0.93 ( 0.82–1.06 )	0.297
BAT_Female	132	Weighted median	0.91 ( 0.82–1.00 )	0.054
BAT_Female	132	Weighted mode	0.90 ( 0.81–1.00 )	0.050
TT_Both	160	IVW	0.98 ( 0.87–1.10 )	0.712
TT_Both	160	MR Egger	1.03 ( 0.84–1.27 )	0.762
TT_Both	160	Weighted median	1.03 ( 0.88–1.21 )	0.707
TT_Both	160	Weighted mode	1.01 ( 0.85–1.20 )	0.902
TT_Male	160	IVW	0.99 ( 0.94–1.04 )	0.653
TT_Male	160	MR Egger	1.00 ( 0.92–1.09 )	0.996
TT_Male	160	Weighted median	0.97 ( 0.91–1.03 )	0.282
TT_Male	160	Weighted mode	0.98 ( 0.92–1.04 )	0.505
TT_Female	196	IVW	0.98 ( 0.93–1.02 )	0.273
TT_Female	196	MR Egger	1.02 ( 0.93–1.12 )	0.672
TT_Female	196	Weighted median	1.00 ( 0.93–1.06 )	0.886
TT_Female	196	Weighted mode	0.97 ( 0.89–1.06 )	0.562
E2_Male	12	IVW	1.01 ( 0.97–1.04 )	0.741
E2_Male	12	MR Egger	0.98 ( 0.88–1.10 )	0.736
E2_Male	12	Weighted median	1.01 ( 0.96–1.06 )	0.756
E2_Male	12	Weighted mode	1.01 ( 0.96–1.07 )	0.751
E2_Female	19	IVW	0.96 ( 0.88–1.04 )	0.307
E2_Female	19	MR Egger	0.96 ( 0.81–1.15 )	0.675
E2_Female	19	Weighted median	0.94 ( 0.87–1.02 )	0.130
E2_Female	19	Weighted mode	0.97 ( 0.88–1.07 )	0.537
FSH_Both	22	IVW	0.99 ( 0.96–1.02 )	0.654
FSH_Both	22	MR Egger	0.97 ( 0.91–1.03 )	0.283
FSH_Both	22	Weighted median	1.02 ( 0.98–1.07 )	0.281
FSH_Both	22	Weighted mode	1.03 ( 0.98–1.08 )	0.252
PRL_Both	15	IVW	1.02 ( 0.98–1.06 )	0.346
PRL_Both	15	MR Egger	0.93 ( 0.85–1.02 )	0.137
PRL_Both	15	Weighted median	1.03 ( 0.97–1.08 )	0.356
PRL_Both	15	Weighted mode	1.03 ( 0.95–1.11 )	0.526
LH_Both	9	IVW	0.99 ( 0.96–1.02 )	0.426
LH_Both	9	MR Egger	0.97 ( 0.92–1.02 )	0.218
LH_Both	9	Weighted median	0.98 ( 0.95–1.01 )	0.223
LH_Both	9	Weighted mode	0.98 ( 0.95–1.01 )	0.210

Abbreviations: E2, Estradiol; FSH, Follicle-Stimulating Hormone; PRL, Prolactin; PROG, Progesterone; TT, Total Testosterone; LH, Luteinizing Hormone; BAT, Bioavailable Testosterone; MR, Mendelian Randomization; SNPs: single nucleotide polymorphisms; OR, odds ratio; IVW: Inverse-variance weighted. CI, confidence interval. Note: *p*-values for the primary analysis (IVW) were corrected for multiple testing using the Bonferroni method to account for 14 independent exposure-outcome comparisons. The adjusted significance threshold was set at *p* < 0.0036 (0.05/14). Results with a raw *p* < 0.05 but ≥ 0.0036 were considered nominally significant/suggestive.

No significant associations were detected between the remaining sex hormone levels, including total testosterone (TT_Both, TT_Male, TT_Female), estradiol (E2_Male, E2_Female), follicle-stimulating hormone (FSH_Both), prolactin (PRL_Both), and luteinizing hormone (LH_Both), and the outcome of depression, using IVW analysis and supplementary analyses. The p-values for all these analyses were greater than 0.05, indicating no evident relationship between these hormone levels and depression risk (Table 1).

Significant heterogeneity was observed in seven exposures, including E2_Female (Q statistic = 44.44, *p* < 0.001), TT_Male (Q statistic = 255.92, *p* < 0.001), TT_Both (Q statistic = 234.58, *p* < 0.001), BAT_Both (Q statistic = 143.90, *p* = 0.002), BAT_Male (Q statistic = 122.79, *p* = 0.002), BAT_Female (Q statistic = 177.60, *p* = 0.004), and TT_Female (Q statistic = 255.56, *p* = 0.002, Table 2). Additionally, significant horizontal pleiotropy was observed in PRL_Both (MR-Egger Intercept = 0.014, *p* value = 0.040, Table 2). The MR-PRESSO analysis identified and excluded outliers for various exposures with depression as the outcome. For BAT_Both, excluding one outlier (rs10745954) did not change the positive result (Outlier-corrected MR-PRESSO result: OR: 0.90, 95% CI: 0.82–1.00, *p* = 0.049), indicating a consistent association with depression. For E2_Female (rs140613202, rs34929649, rs734518), TT_Both (rs11830764), and TT_Male (rs7314285), excluding the identified outliers did not alter the results, which remained negative, indicating no association with depression (Table 3). The stability of our finding was validated by a leave-one-out analysis, which confirmed that excluding any single SNP would not significantly impact the observed association (Fig. 2C and 3C). Additionally, funnel plots indicated balanced pleiotropy, further supporting the robustness of the results (Fig. 2D and 3D).

**Table 2.** **Results of heterogeneity test and pleiotropy test for instrumental variables**.

Exposure	Heterogeneity		Pleiotropy	
	Q statistic (IVW)	*p *value	MR-Egger Intercept	*p* value
E2_Male	4.96	0.933	0.004	0.640
FSH_Both	28.70	0.121	0.006	0.321
E2_Female	44.44	< 0.001	–0.001	0.954
PRL_Both	9.99	0.763	0.014	0.040
PROG_Male	10.46	0.107	0.007	0.694
TT_Male	255.92	< 0.001	0.000	0.753
LH_Both	10.64	0.223	0.010	0.312
PROG_Both	20.44	0.555	–0.005	0.477
TT_Female	255.56	0.002	–0.002	0.285
BAT_Both	143.90	0.002	0.001	0.633
PROG_Female	28.69	0.534	0.008	0.304
BAT_Male	122.79	0.002	0.003	0.190
BAT_Female	177.60	0.004	0.000	0.932
TT_Both	234.58	< 0.001	–0.001	0.548

E2, Estradiol; FSH, Follicle-Stimulating Hormone; PRL, Prolactin; PROG, Progesterone; TT, Total Testosterone; LH, Luteinizing Hormone; BAT, Bioavailable Testosterone; MR, mendelian randomization; IVW: Inverse-variance weighted.

**Table 3.** **MR-PRESSO pleiotropy analysis**.

Exposure	Raw		Outlier corrected		Global *p*	outliers	Distortion *p*
	OR (95% CI)	*p*	OR (95% CI)	*p*			
BAT_Both	0.90 (0.82–0.99)	0.035	0.90 (0.82–1.00)	0.049	0.006	rs10745954	0.817
BAT_Female	0.93 (0.87–1.00)	0.038	NA (NA–NA)		0.001	NA	NA
BAT_Male	1.00 (0.93–1.07)	0.956	NA (NA–NA)		0.007	NA	NA
E2_Female	0.96 (0.88–1.04)	0.321	0.98 (0.92–1.04)	0.455	<0.001	rs140613202, rs34929649, rs734518	0.135
E2_Male	1.01 (0.98–1.03)	0.633	NA (NA–NA)		0.935	NA	NA
FSH_Both	0.99 (0.96–1.02)	0.659	NA (NA–NA)		0.134	NA	NA
LH_Both	0.99 (0.96–1.02)	0.449	NA (NA–NA)		0.352	NA	NA
PRL_Both	1.02 (0.98–1.06)	0.283	NA (NA–NA)		0.768	NA	NA
PROG_Both	0.95 (0.92–0.98)	0.004	NA (NA–NA)		0.579	NA	NA
PROG_Female	1.01 (0.99–1.02)	0.342	NA (NA–NA)		0.534	NA	NA
PROG_Male	0.96 (0.88–1.06)	0.475	NA (NA–NA)		0.141	NA	NA
TT_Both	0.98 (0.88–1.09)	0.710	0.97 (0.88v1.08)	0.618	<0.001	rs11830764	0.912
TT_Female	0.98 (0.94–1.02)	0.371	NA (NA–NA)		<0.001	NA	NA
TT_Male	0.99 (0.94–1.04)	0.632	0.99 (0.95–1.04)	0.714	<0.001	rs7314285	0.851

E2, Estradiol; FSH, Follicle-Stimulating Hormone; PRL, Prolactin; PROG, Progesterone; TT, Total Testosterone; LH, Luteinizing Hormone; BAT, Bioavailable Testosterone; MR, mendelian Randomization, OR: odds ratio; CI, confidence interval.

## Discussion

This MR analysis identified significant associations between PROG_Both, BAT_Both, and depression. However, these associations became non-significant when stratified by gender, indicating no evident relationship between PROG or BAT levels and depression in either males or females. No significant associations were found between other sex hormones, including TT, E2, FSH, PRL, and LH, and depression, suggesting that these hormones do not significantly impact depression risk. MR-Egger regression indicated horizontal pleiotropy in PRL_Both. The research results were validated for reliability and robustness through leave-one-out analysis and balanced pleiotropy, as shown in funnel plots.

Earlier observational research has suggested a possible link between sex hormones and depression. PROG and its metabolites, particularly during significant hormonal fluctuations such as pregnancy and the menstrual cycle, have been shown to influence mood and contribute to depressive symptoms. PROG, a steroid hormone, performs a critical function in the monthly menstrual cycle, pregnancy, and embryonic development. Andrade *et al*. [31] found that low-dose PROG has a gender-dependent antidepressant effect in rats, with chronic administration of low-dose PROG reversing depressive-like behaviors in female rats, which is consistent with our findings. This was supported by mechanisms involving gut microbiota. Sovijit *et al*. [32] suggested that ovarian PROG alleviates depressive and anxiety-like behaviors by increasing the number of lactobacilli in the gut microbiota of ovariectomized mice, thereby linking PROG’s mood-regulating effects with gut microbiota composition. However, interestingly, contrary to our findings, Murphy *et al*. [33] found that PROG and its 5-alpha reduced metabolite, 5 alpha-dihydroprogesterone, which rise significantly during pregnancy, are linked to mood changes. Elevated levels of these metabolites were found to be strongly associated with symptoms of depression during the latter half of pregnancy. BAT refers to the fraction of testosterone in the bloodstream that is readily available to tissues, which is considered a more accurate indicator of a man’s androgen status compared to TT. Chen *et al*. [34] found that lower levels of BAT, as indicated by the free testosterone index (FTI), are significantly associated with increased symptoms of depression in adult men. Depressed men had lower average BAT levels than the non-depressed group. The prevalence of biochemical hypogonadism was also higher among depressed men, suggesting that lower BAT levels might be a risk factor for depression in middle-aged men [11]. The MR study suggests that there is a genetic linkage between PROG, BAT, and depression.

Additionally, further exploration is required to understand the specific mechanisms by which PROG and BAT influence depression and to develop corresponding therapeutic strategies. The discrepancy between significant overall associations and non-significant sex-stratified results is likely due to reduced statistical power in stratified cohorts. For example, the inclusion of only 7 SNPs for PROG_Male increases the risk of Type II error, indicating that the current data may be insufficient to detect a true causal effect, rather than proving its absence. To explain the identified causal links, several molecular mechanisms are proposed. First, PROG acts as a precursor to neurosteroids like allopregnanolone, which functions as a potent positive allosteric modulator of GABA-A receptors to exert antidepressant effects—a mechanism recently validated by the clinical success of GABA-targeted therapies [35]. Second, both PROG and BAT are involved in neuroimmunomodulation; they can suppress neuroinflammation by inhibiting microglia overactivation, reducing pro-inflammatory cytokine levels, and thereby protecting hippocampal neurons [36]. Third, BAT may stabilize mood by antagonizing the hypothalamic-pituitary-adrenal (HPA) axis stress response [37]. Integrating these neuroendocrine pathways with the gut-brain axis provides a comprehensive framework for understanding hormone-related depression. However, when the results were stratified by gender, no genetic correlation was observed. Sample size limitations of PROG in gender-stratified analyses may reduce the statistical power, making it difficult to detect genetic associations. The lack of significant associations following sex stratification is likely attributable to the reduced sample size and the limited number of available instrumental variables for gender-specific analyses. For instance, the inclusion of only 7 SNPs for PROG_Male significantly diminishes the statistical power to detect true associations. This limitation increases the risk of Type II errors (false negatives), suggesting that these non-significant results may reflect an insufficient capacity to detect effects rather than the definitive absence of sex-specific potential causal associations. Further research needs to analyze larger samples of GWAS data to confirm these findings. The inconsistency in the results might be due to the fact that the hormone levels themselves are not related to the outcome, but rather the ratios of different hormones that may influence it. This highlights the importance of further investigating the impact of sex hormone ratios on depression. Additionally, further exploration is required to understand the specific mechanisms by which PROG and BAT influence depression and to develop corresponding therapeutic strategies.

To further elucidate the causal links identified in this MR study, several molecular mechanisms can be proposed as verifiable hypotheses. The antidepressant potential of PROG’s may stem from its metabolites, such as allopregnanolone, which act as positive allosteric modulators of GABA-A receptors to alleviate anxiety and depressive symptoms [38]. Moreover, both PROG and BAT may play a role in neuroimmunomodulation; sex hormones have been observed to suppress neuroinflammation by inhibiting microglial activation and reducing the release of pro-inflammatory cytokines [36]. Regarding BAT, the regulation of the hypothalamic-pituitary-adrenal (HPA) axis stress response may be a key pathway through which testosterone helps maintains emotional stability [39]. Integrating these neurotransmitter and neuroendocrine pathways with the gut-brain axis provides a more holistic framework for understanding the biological basis of sex hormone-related depression [40]. Importantly, a clear distinction must be made between the genetically determined lifelong exposure captured by this MR study and the short-term clinical or physiological hormonal fluctuations encountered in medical practice. MR estimates reflect the cumulative, lifelong impact of genetically determined average hormone levels on the risk of depression, representing a stable biological baseline. This differs fundamentally from acute hormonal shifts, such as the rapid declines in PROG and estrogen during the postpartum period or cyclical variations during the menstrual cycle, which are often primary triggers for clinical depressive episodes. While our findings underscore the causal importance of steady-state hormonal equilibrium, the biological pathways through which acute fluctuations induce mood disorders may involve distinct mechanisms. Therefore, these results should be interpreted as highlighting the influence of long-term hormonal stability rather than the immediate effects of transient physiological transitions.

LH is a crucial hormone in the human endocrine system, which is produced by the anterior pituitary gland, and it works in conjunction with FSH. When compared to women in the control group, women with depression exhibited LH pulses with slower disordered frequency-, indicating dysregulation of the hypothalamic-pituitary-gonadal axis in severe depression [41]. The blood FSH levels were significantly lower in female patients with major depressive disorder who had current suicidal ideation or attempts compared to those without such ideation or attempts [42]. E2 is a potent estrogen steroid hormone and the primary female sex hormone. E2 regulates the expression of genes involved in serotonin neurotransmission, which may influence mood and contribute to depression. Hernández-Hernández *et al*. [43] suggested that E2 can increase the expression of serotonin transporters and decrease serotonin receptor expression, potentially explaining its mood-stabilizing effects. PRL is a peptide hormone secreted mainly by the adenohypophysis, playing a key role in numerous physiological processes, particularly in reproduction and lactation. The PRL response to serotonin stimulation was significantly attenuated in patients with major depression, indicating an impairment of serotonin function in depression rather than a pituitary abnormality [44]. TT refers to the sum of all testosterone present in the blood, including free testosterone and bound testosterone. Low testosterone was weakly associated with depression in men, while no consistent associations were observed in women [12]. Although previous observational studies have reported associations between these sex hormones and depression, this MR study found no potential causal association between LH, E2, PRL, and TT and depression, even when analyzed separately by gender. Several reasons could explain this inconsistency. Firstly, observational studies may be subject to confounding variables that can distort the true relationship between hormones and depression. Secondly, the MR study might have limitations in sample size or statistical power, especially when stratifying by gender. Larger sample sizes in future studies could provide more definitive insights.

This MR study has several limitations. First, this study primarily utilized genetic variants identified in GWAS conducted in European populations, raising concerns about the universality and generalizability of the results across populations with different genetic backgrounds. Second, despite the study’s focus on the link between sex hormones and depression, the etiology and pathophysiology of depression are multifactorial, potentially involving interactions between genetic, environmental, and lifestyle factors. Third, while the study identified a causal linkage between sex hormones and depression, further investigation is required to clarify the precise mechanisms underlying this potential causal association. Fourth, although seven major sex hormones were included, this study may not be fully comprehensive. Other hormones and neurosteroids potentially linked to depression, such as dehydroepiandrosterone (DHEA) or specific hormone precursors, were not analyzed due to the current lack of publicly available, large-scale GWAS summary data. As more genetic datasets become available, future research should aim to incorporate a broader range of hormonal exposures to provide a more complete picture. Fifth, there is a potential for bias due to the inconsistency in gender composition between the exposure and outcome datasets. While some sex hormone exposures used sex-stratified GWAS data, others only provided mixed-gender summary statistics. Conversely, the outcome data for depression from the FinnGen collaboration was primarily derived from a mixed-gender population. Using sex-specific genetic instruments against a mixed-gender outcome, or vice versa, may lead to an underestimation or overestimation of causal effects and limit our ability to fully elucidate sex-specific biological pathways. Sixth, the use of inconsistent *p*-value thresholds (*p* < 5 × 10^-8^ and *p* < 5 × 10^-6^) for different hormones may influence the comparability of our findings. While relaxing the threshold for certain hormones provided the necessary instruments to conduct the analysis, it may have introduced variants with relatively weaker associations compared to those meeting the stricter threshold. This discrepancy could potentially affect the precision of the causal estimates and should be considered when comparing the relative effect sizes across different sex hormones.

## Conclusions

In summary, this MR study suggests that genetically predicted PROG levels may serve as a protective factor against the risk of depression. A potential suggestive association was also observed for BAT, although it did not maintain significance after Bonferroni correction. These findings highlight the importance of long-term hormonal equilibrium in the etiology of depression, distinct from the mechanisms of acute physiological fluctuations. Future longitudinal studies and experimental research are needed to further elucidate the biological pathways through which sustained hormonal levels influence mental health.

## Availability of Data and Materials

The data that support the findings of this study are available from the corresponding author.

## Supplementary Material

Supplementary material associated with this article can be found, in the online version, at https://doi.org/10.62641/aep.v54i4.2093.
