# Supplementary material for: Exploring the Causal Relationship Between Sex Hormones and Depression by Means of Two-Sample Mendelian Randomization Analysis
Source: Actas Esp Psiquiatr. 2026 Aug 15;54(4):1245–56. doi: 10.62641/aep.v54i4.2093 (PMC13571192; doi:10.62641/aep.v54i4.2093)
Supplement: Supplementary file 1 [file ActEsp-54-4-1245-1256-s1.zip › Supplemental Table 1.docx]

Supplemental Table 1. The detailed summary of GWAS datasets in MR analysis

| Traits | Sex | Data sources | **GWAS ID** | Sample size  (cases/controls) | Ancestry | PMID |
| --- | --- | --- | --- | --- | --- | --- |
| FSH | Both | IEU | GCST90241193 | 3,301 | EUR | 29875488 |
| LH | Both | IEU | GCST90241825 | 3,301 | EUR | 29875488 |
| E2 | Male | GWAS Catalog | GCST90020091 | 147,690 | EUR | 34255042 |
| E2 | Female | GWAS Catalog | GCST90020092 | 163,985 | EUR | 34255042 |
| PROG | Both | Leipzig Health Atlas |  | 2,070 | EUR | 31169883 |
| PROG | Male | Leipzig Health Atlas |  | 1,358 | EUR | 31169883 |
| PROG | Female | Leipzig Health Atlas |  | 1,261 | EUR | 31169883 |
| PRL | Both | GWAS Catalog | GCST90012030 | 21758 | EUR | 33067605 |
| BAT | Both | GWAS Catalog | GCST90012104 | 382,988 | EUR | 32042192 |
| BAT | Male | GWAS Catalog | GCST90012103 | 178,782 | EUR | 32042192 |
| BAT | Female | GWAS Catalog | GCST90012102 | 188,507 | EUR | 32042192 |
| TT | Both | GWAS Catalog | GCST90012114 | 425,097 | EUR | 32042192 |
| TT | Male | GWAS Catalog | GCST90012113 | 194,453 | EUR | 32042192 |
| TT | Female | GWAS Catalog | GCST90012112 | 230,454 | EUR | 32042192 |

MR, Mendelian Randomization; SNPs: single nucleotide polymorphisms;
